# The role of psychopathological symptoms in lumbar stenosis: A prediction model of disability after lumbar decompression and fusion

**DOI:** 10.3389/fpsyg.2023.1070205

**Published:** 2023-03-22

**Authors:** Edoardo Mazzucchi, Giuseppe La Rocca, Davide Cusumano, Paola Bazzu, Fabrizio Pignotti, Gianluca Galieri, Pierluigi Rinaldi, Vincenzo De Santis, Giovanni Sabatino

**Affiliations:** ^1^Institute of Neurosurgery, Fondazione Policlinico Universitario A. Gemelli IRCCS, Catholic University, Rome, Italy; ^2^Unit of Neurosurgery, Mater Olbia Hospital, Olbia, Italy; ^3^Unit of Medical Physics, Mater Olbia Hospital, Olbia, Italy; ^4^Clinical Psychology Service, Mater Olbia Hospital, Olbia, Italy; ^5^Unit of Radiology, Mater Olbia Hospital, Olbia, Italy; ^6^Unit of Orthopedics, Mater Olbia Hospital, Olbia, Italy

**Keywords:** lumbo-sacral instrumentation, minimally invasive spine surgery, depression, obsessivity, anxiety, bio-psychosocial model

## Abstract

**Introduction:**

Pre-operative psychological factors may influence outcome after spine surgery. The identification of patients at risk of persisting disability may be useful for patient selection and possibly to improve treatment outcome.

**Methods:**

Patients with neurogenic claudication associated with degenerative lumbar spinal stenosis (DLSS) performed a psychological assessment before lumbar decompression and fusion (LDF) surgery. The following tests were administrated: Visual Analogic Scale; Symptom Checklist-90 (SCL-90-R), Short Form-36 and Oswestry Disability Index (ODI). The primary outcome was ODI score lower than 20. A cross correlation matrix (CCM) was carried out with significant variables after univariate analysis and a linear logistic regression model was calculated considering the most significant variable.

**Results:**

125 patient (61 men and 64 women) were included in the study. Seven parameters of the SCL-90-R scale showed statistical significance at the univariate analysis: obsessivity (*p* < 0.001), Current Symptom Index (*p* = 0.001), Global Severity Index (*p* < 0.001), depression (*p* < 0.001), positive Symptom Total (*p* = 0.002), somatization (*p* = 0.001) and anxiety (*p* = 0.036). Obsessivity was correlated with other significant parameters, except GSI (Pearson’s correlation coefficient = 0.11).

The ROC curve for the logistic model considering obsessivity as risk factor, has an area under the curve of 0.75.

**Conclusion:**

Pre-operative psychopathological symptoms can predict persistence of disability after LDF for DLSS. Future studies will evaluate the possibility of modifying post operative outcome through targeted treatment for psychological features emerged during pre-operative assessment.

## Introduction

Degenerative lumbar spinal stenosis (DLSS) is a common condition that may produce back pain, pain radiating to lower limbs, neurogenic claudication. Decompression surgery with fusion has been proposed as a possible treatment for symptomatic patients with signs of instability (Resnick et al., 2014).

Despite scientific and technological advancements, unsatisfying outcome is relatively common (Hébert et al., 2020). Pain and disability are generally considered as the most important outcome variables, but both are the result of the combination of multiple components, not necessarily related to anatomical conditions or surgical techniques.

Pain is a complex symptom which results from the combination of multiple components: nociceptive, neuropathic, psychological and social features. Persistence of pain after surgery in general (not only spine surgery) showed to be related to several risk factors, like psychological status, fear of movement, executive functions, for example (Feinmann et al., 1987; Ghoneim and O’Hara, 2016; Giusti et al., 2020). Post-operative pain may in turn worsen disability through fear of movement beliefs and pain catastrophizing mechanisms (Archer et al., 2011; Varallo et al., 2022). Depression and anxiety are particularly common in patient with back pain (Sinikallio et al., 2011; Falavigna et al., 2012) and they have been hypothesized in previous studies to negatively influence outcome in spine surgery (Dobran et al., 2018). On the other hand, pain and limitation of autonomy, which are typical symptoms of DLSS, may generate depression, with a complex interplay between anatomical condition (stenosis), neurological function and psychological features.

An evaluation of bio-psychosocial risk factors has already been performed in patients undergoing surgical treatment for lumbar disk herniation, revealing that the level of education, work satisfaction, duration of sick leave, passive-avoidance coping function, low expectations on work return and fear of movement before surgery are for example to be evaluated as risk factors for pain or disability (den Boer et al., 2006; Johansson et al., 2010). Fear-avoidance beliefs have been associated with pre-operative function and post-operative outcome in patients with lumbar stenosis in previous studies (Burgstaller et al., 2017; Wada et al., 2021; Minetama et al., 2022).

The identification of patients at higher risk for unsatisfying outcome after surgery may guide targeted treatments to improve mental health before or after surgery (Rolving et al., 2015). For example, patient education has reduced the level of anxiety in patients undergoing spine surgery (Strøm et al., 2018).

Aim of the present study is investigating if a pre-operative psychological assessment may be able to predict disability outcome in a cohort of patients who undergo lumbar decompression and fusion (LDF).

## Methods

We included consecutive patients who underwent LDF surgery for DLSS in a single neurosurgical center in 18 months. Patients were prospectively followed for at least 1 year.

All patients signed a written informed consent and the study was previously approved by the local Ethics Committee, protocol number 276/2020/CE.

### Inclusion criteria

- Radiological diagnosis of DLSS
- Signs and symptoms of instability (Resnick et al., 2014)
- Neurogenic claudication
- More than 6 months of physical therapy/pain therapy treatment without efficacy
- Age between 18 and 80 years
- Consent to participate in the study

### Exclusion criteria

- Previous or actual treatment for anxiety or depression
- Psychotherapy in the last 2 years
- Osteoporosis
- Neurotoxic chemotherapy
- Other causes of chronic pain
- Active neoplastic disease
- Cognitive impairment

### Psychological and functional-disability assessment

Patients with indication for LDF who consented to participate in the study, underwent a psychological and functional impairment assessment. The following scales have been administrated before surgery:

- Visual Analogic Scale (VAS) both for Back Pain (VAS-BP) and for Leg Pain (VAS-LP), ranging from 0 to 10, in which a lower score demonstrates less pain (Haefeli and Elfering, 2006)
- The Symptom Checklist 90-R (SCL90-R) (Derogatis, 1992; Schmitz et al., 1999; Derogatis, 2014)
- Oswestry Disability Index (ODI)(Monticone et al., 2009)
- Short Form 36 (SF-36) a 36-item self-administrated survey on patient health (Ware and Sherbourne, 1992)

All scales except SCL90-R were readministered at follow-up.

SCL90-R is a self-reported assessment tool used to determine the number of psychological symptoms in order to define psychopathological dimensions. It includes 90 items subdivided into nine subscales:

- Somatization: the discomfort related to perception of body disfunctions
- Obsessivity: persisting and compelling thoughts, drives or actions
- Interpersonal sensitivity: feelings of inadequacy and inferiority
- Depression: desperation, suicidal thoughts and cognitive and somatic symptoms related to depression
- Anxiety: nervousness, tension, tremors, panic attacks
- Hostility: thought, feelings and behaviors related to rage
- Phobic anxiety: persistent fear reaction to a specific situation, perceived as irrational or disproportioned
- Paranoid ideation: hostility, suspiciousness, grandiosity, delirium
- Psychoticism: withdrawal, isolation and schizophrenic symptoms

Each of the subscales includes 6–13 items, and the score of each dimension is calculated as the mean of the scores of all the items included, which refer to symptoms reported during the previous week. Moreover, three global indices are computed: Global Severity Index (GSI), which measures overall psychological distress; Positive Symptom Distress Index (PSDI), a measure of the intensity of symptoms, and Positive Symptom Total (PST) that represents the number of self-reported symptoms. We also recorded a Current Symptom Index (CSI), defined as the mean value of Somatization, Obsessivity, Depression, Anxiety, Phobic anxiety, and Psychoticism (Unoka et al., 2022). A cut off to define pathologic values for the studied population (spine surgery patients) is not available, so we considered raw scores in our analysis.

### Surgical treatment

All the surgical interventions were carried out at the same Institution by the same surgeons. All procedures were performed on a TruSystem^®^ 7000 table (TRUMPF^®^ Medizin Systeme GmbH) with a percutaneous technique for pedicular screw placement guided by fluoroscopy or CT-based navigation. CT-based procedures were carried out with a BrainLab Curve 1.2^®^ navigation system (Brainlab AG^®^, Munich, Germany) linked to AIRO Mobile intraoperative CT scan (Brainlab AG^®^, Munich, Germany). Instrumentation systems are manufactured by NuVasive^®^ (San Diego, California, United States). Intra operative Neuro-monitoring (IOM) Nerve Monitor System (NVM5^®^) was provided by NuVasive® and was used for each case (La Rocca et al., 2022). Interbody fusion was performed only in selected cases. After screw placement a laminectomy with flavectomy and lateral recess decompression was performed. A drainage tube was positioned in all cases and removed the day after surgery when the patient was mobilized.

Clinical and radiological data were registered for each patient. Moreover, we recorded surgical time and the accuracy of screw placement was evaluated blindly by a senior neuroradiologist on the CT scan performed the day after the procedure, following the Gertzbein-Robbins scale (Gertzbein and Robbins, 1990).

Primary outcome was considered an ODI lower than 20 at follow up, reflecting minimal or no disability.

### Statistical analysis

The predictive performance of the clinical and psychological parameters in identifying the clinical end-points at the univariate analysis was assessed using the Wilcoxon-Mann Whitney or the t-test, depending on the normality of the data distribution with respect to the considered outcome, which was previously assessed using the Shapiro–Wilk test (Taylor, 1997; Cusumano et al., 2021).

The Benjamini-Hochberg method was adopted to adjust the value of p-values obtained from the Wilcoxon test and compensating for the issue of multiple comparisons (McHugh, 2011).

A cross-correlation matrix was carried out among the variables showing significance at the univariate analysis, considering Pearson’s correlation coefficient (PCC) as correlation metric (Chan, 2003). Parameters with PCC < |0.3|were considered as not correlated. A linear logistic regression model was calculated considering the most significant variable at the univariate analysis (Fleiss et al., 2013).

The Receiver Operating Characteristic (ROC) curve was calculated for the model, and the value of the area under the curve (AUC) was determined, using a bootstrap technique with 2000 samples to identify the 95% confidence interval (International Commissioning on Radiation Units and Measurements, 2008).

The best cut-off value was determined calculating the Youden Index and the values of sensitivity, specificity, negative and positive predictive values were evaluated at that point.

Considering the absence of an external validation set, the reliability of the model elaborated was evaluated by means of a 10-folds cross-validation analysis with five iterations (Cusumano et al., 2021, 2022).

The entire statistical analysis and processing was performed using R software and dedicated packages (R Core Team version, Wien, Austria; Robin et al., 2011; Gatta et al., 2018).

The list of the variables included in the statistical analysis is available in Supplementary material. Acquisition and analysis of data was performed blindly by different researchers.

## Results

### Clinical data

One hundred forty-seven patients underwent LDF in the period of the study. Due to incomplete follow up, 22 patients were excluded from the cohort. One hundred twenty-five patient (61 men and 64 women) were included in the statistical analysis. The median age was 61 years (23–78). Median length of stay was 2 days (Feinmann et al., 1987; Archer et al., 2011; Resnick et al., 2014; Ghoneim and O’Hara, 2016; Giusti et al., 2020; Hébert et al., 2020). According to Gertzbein-Robbins scale 14 (2.3%) screws were misplaced (4 screws was classified as grade D and 10 grade E). Nevertheless, none of these patients had clinical signs of radiculopathy.

Clinical and radiological data are described in Tables 1–5.

**Table 1:** Demographic and clinical data. Data are displayed as median (minimum – maximum) when appropriated.

Variable	
Sex (M:F)	61:64
Age (years)	61 (23–78)
BMI (kg/m^2^)	27 (17.6–42.1)
Smoke (yes:no)	50:75
Length of stay (days)	2 (1–6)
Follow-up (days)	594 (385–937)

M, male; F, female; BMI, body mass index.

**Table 2:** Surgical data. Data are displayed as median (minimum – maximum) when appropriated.

Surgical data	
Screws per patient	4 (2–8)
Previous surgery (yes:no)	31:94
TLIF (yes:no)	69:56
Complications (yes:no)	8:117
Operation time (min)	145 (55–385)

TLIF, transforaminal lumbar interbody fusion.

**Table 3:** Surgical data. Operated levels.

Location	Patients treated
L3L4	11
L4L5	41
L5S1	30
L2L4	3
L3L5	26
L4S1	12
L2L5	6
L3S1	2

**Table 4:** Clinical outcome data. Pain and disability before (pre) and after (post) surgical intervention, as measured with Visual Analogic Scale and Oswestry Disability Index.

Variable	
VAS LP pre	8 (4–10)
VAS LP post	2 (0–10)
VAS BP pre	8 (5–10)
VAS BP post	3 (0–10)
ODI pre	56 (16–90)
ODI post	30 (0–80)
ODI good outcome (yes:no)	71:54

Good outcome for Oswestry Disability Index is defined as lower than 20 (see text). VAS LP, Visual Analogic Scale Leg Pain; VAS BP, Visual Analogic Scale Back Pain; ODI, Oswestry Disability Scale.

**Table 5:** Radiological outcome data.

Gertzbein Robbins	
A	542
B	38
C	10
D	4
E	10

Classification of pedicle screw placement (Gertzbein-Robbins classification, see text for details).

The rate of good outcome (i.e., ODI < 20 at follow up) was 56.8%.Patient experienced a significant improvement in ODI (*p* < 0.001), VAS-LP (*p* < 0.001) and VAS-BP (*p* < 0.001). The following complication were encountered: intraoperative screw mispositioning (1), unintended dural opening (3), Transforaminal Lumbar Interbody Fusion (TLIF) subsidence (1), postoperative anemization (1), post-operative hematoma (1), surgical wound dehiscence (1).

A total of seven parameters showed statistical significance at the univariate analysis in predicting good disability outcome (ODI < 20): SCL-90-R obsessivity symptoms subscale (*p* < 0.001), SCL-90-R depression symptoms subscale (*p* < 0.001), Current Symptom Index (*p* < 0.001), Global Severity Index (*p* < 0.001), Positive Symptom Total (*p* = 0.002), somatization symptoms subscale (*p* = 0.001) and anxiety (*p* = 0.036). They are reported in Table 6, together with the p-values obtained after the application of Benjamin-Hoch correction.

**Table 6:** Significant parameters able to predict good disability outcome (ODI < 20) at the univariate analysis.

Clinical parameter	*p*-value
Obsessivity	<0.001
CSI	<0.001
GSI	<0.001
Depression	<0.001
PST	0.002
Somatization	0.001
Anxiety	0.036

CSI, Current Symptom Index; GSI, Global Severity Index; PST, Positive Symptom Total.

**Figure 1:**
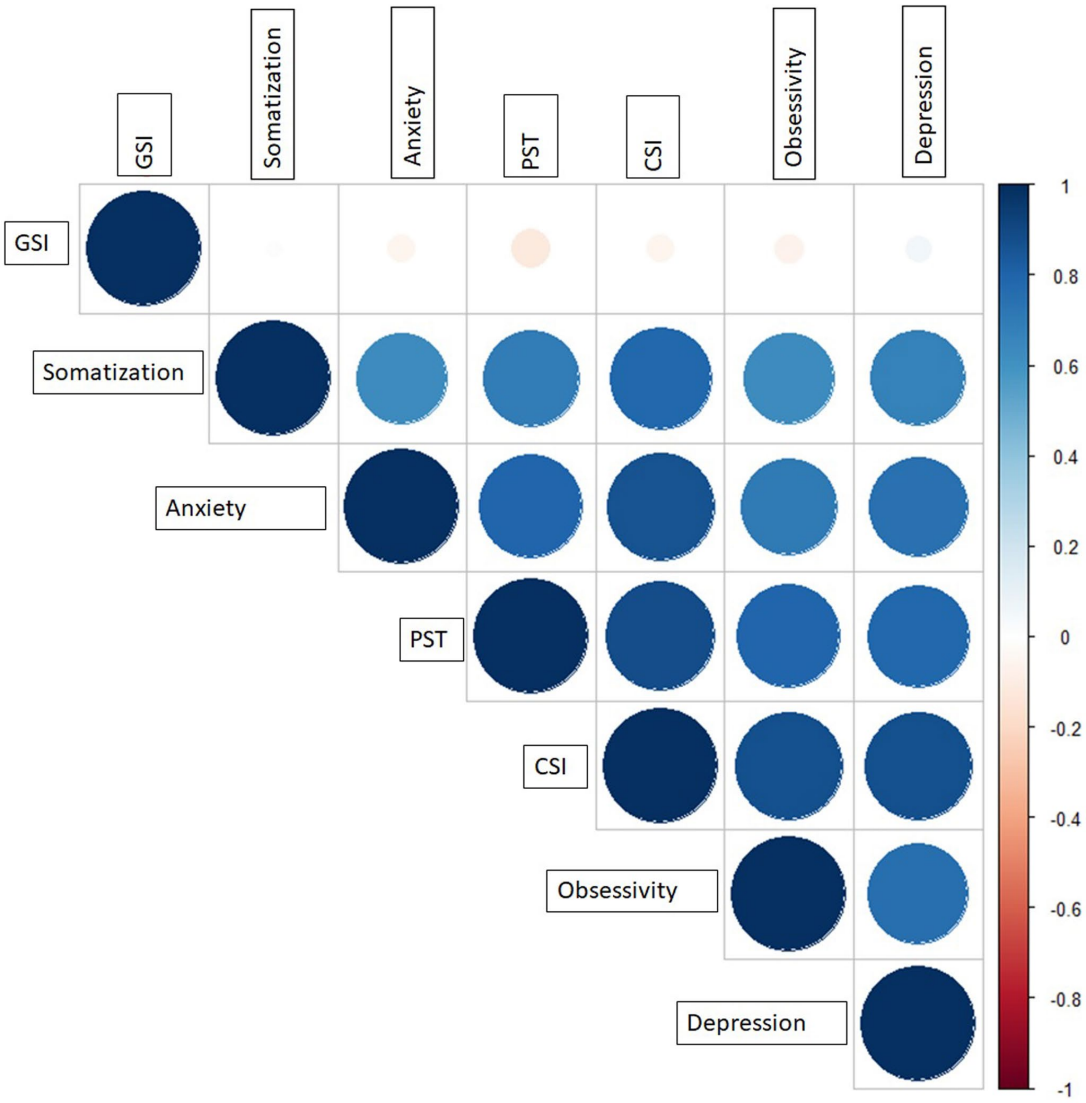
Cross correlation matrix among the significant features at the univariate analysis. CSI, Current Symptom Index; GSI, Global Severity Index; PST, Positive Symptom Total.

Cross validation matrix calculated among the significant parameters is reported in Figure 1: the most significant feature, obsessivity, resulted to be correlated with all the others, except to GSI where a PCC equal to 0.11 was observed.

**Figure 2:**
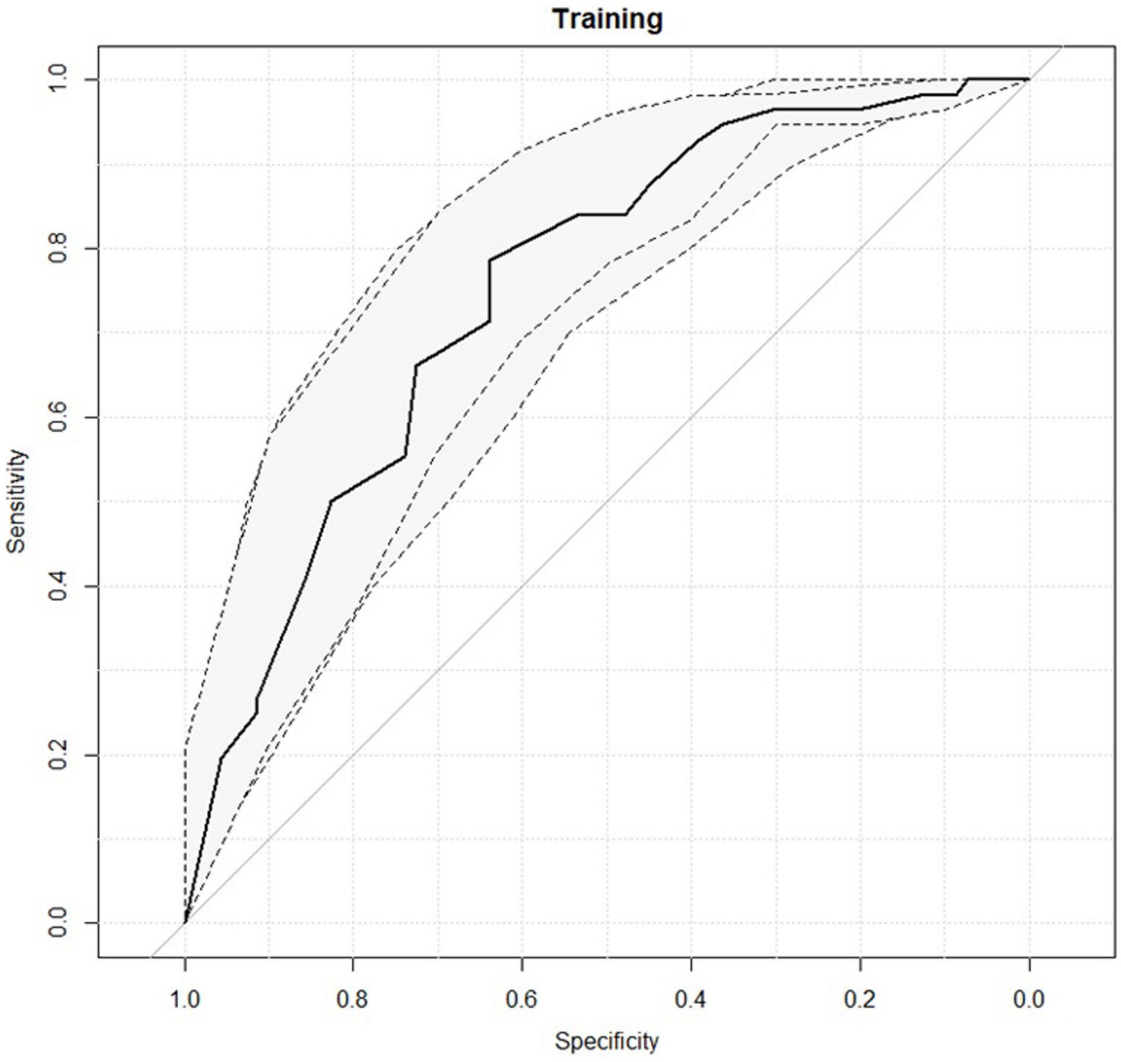
Receiver operating characteristic (ROC) curves for the predictive model with the 95% confidence intervals reported in grey.

Figure 2 reports the ROC curves of the predictive model obtained considering obsessivity symptoms subscale as single variable.

The predictive performance of the logistic model at the best cut-off threshold is reported in Table 7. An AUC value of 0.75 was observed with an interval of confidence ranging from 0.66 and 0.83.

**Table 7:** Predictive performance of the logistic model (see text for details).

	Sensitivity	Specificity	Threshold	J_index	AUC
Training	78.57	63.77	0.42	0.42	0.75 (0.66–0.83)

The values of posterior predictive check (PPC) are reported in Supplementary Table S1.

Combining the obsessivity with the GSI no significant improvement in predictive performance was observed: an AUC of 0.77 was observed.

The predictive model with one single variable was also evaluate in 10-folds cross validation, obtaining an AUC of 0.75 (0.60–0.90 as 95% confidence interval).

## Discussion

The present study showed how clinical outcome after LDF may be predicted through psychopathological symptoms evaluation. In particular, univariate analysis shows association with GSI, PST, CSI, depression, somatization, anxiety and obsessivity symptoms subscales of the SCL-90-R questionnaire (see Table 6). Previous study demonstrated an association between depression and outcome after fusion and non-fusion surgery for DLSS (Sinikallio et al., 2009, 2011; McKillop et al., 2014; Held et al., 2022). In patient affected from DLSS who already failed conservative treatments, limitation of physical activity and pain may cause depression, (Wahlman et al., 2014; Strøm et al., 2018) in particular back pain (Pinheiro et al., 2016). Indeed, there is a bidirectional relationship between depression and disability in spinal degenerative pathology before surgery (Sinikallio et al., 2011). Depression can also negatively influence rehabilitation after surgery (Ghoneim and O’Hara, 2016). It is associated with higher cumulative opioid use, complications, readmission and cost (O’Connell et al., 2018). It should also be noted that psychological stress has an immunosuppressive effect that may result in an increased risk for complications, (Starkweather et al., 2006) even if this was not significant in our case series. Somatization has already been associated with depression in a population of female patient with lumbar stenosis (Kaptan et al., 2012).

To our knowledge, the association of obsessivity symptoms with disability outcome has never been described previously. The use of SCL-90-R questionnaire in patients affected from low back pain has been already described in several studies (Schiphorst Preuper et al., 2007) but its use in relation with neurosurgical treatment for DLSS has been applied rarely (Dobran et al., 2018). Obsessivity, mainly as a subclinical disorder, may be associated with chronic pain, especially low-back pain (Henry et al., 2011; Mehraban et al., 2014). The patient may have selective attention to pain related stimuli, (Karno et al., 1988) and adopt pain avoidance behaviour (Pfingsten et al., 2001) in the post-operative period, with worse disability outcome. On the other hand, pain may be a distraction from emotional distress, some patients with obsessive compulsive disorder may desire to preserve pain to control psychological suffering (Hezel et al., 2012).

The proposed model showed a high capacity of predicting disability outcome in patient undergoing LDF for DLSS. Psychological and psychopathological symptoms assessment should be, in our opinion, part of the diagnostic framework of patients for whom a LDF is indicated on the basis of neurological symptoms and radiological data. As a matter of fact, in our study, psychological features, such as presence of obsessivity or depression symptoms showed a stronger correlation with outcome than other clinical factors which are commonly included in the preoperative workout to define the risk of inefficacy of surgery such as age, smoke, BMI and so on.

A pre-operative complementary psychological assessment may provide several advantages both for the patient and for the physician in the clinical practice. First, we may use these additional data to improve information of patient before surgery: the patient has various treatment options and both the patient and the treating physician should be aware that, considering his/her psychological pre-operative profile, the probability of persisting disability is higher. Moreover, information may greatly improve the capacity of patient to cope with disease, reduce pre-operative anxiety, induce positive attitudes (Strøm et al., 2018). The possibility of a pre-operative treatment trial for previously unrecognized psychological problems should also be evaluated, considering that intervention for DLSS can normally be postponed for a few months without significant adjunctive risk for the patient. We have already started considering this option in our clinical practice; this will be object of future studies. Nevertheless, the improvement of measures of clinical outcome after surgery is significant also in patients with depression: this means that depression is a relevant factor in clinical outcome, but also depressed patients or patients with higher obsessivity score in the SCL-90-R questionnaire have a statistically significant improvement.

We evaluated the pedicle screw placement with the Gertzbein-Robbins scale, which provided results in line with previous studies (Gelalis et al., 2012). The rate of complications and the length of stay are other indices that the surgical procedure and perioperative management have been performed accurately. The integration of different members of the healthcare team results in close communication between experts in distinct methodologies of treating chronic pain and promotes a biopsychosocial approach to the patient’s pain (Miller et al., 2005). Rather than relying solely on biophysical perspectives and intervention, an integrated, biopsychosocial approach for evaluation of patients and management of their symptoms has proven to be an effective strategy for symptom relief and control, if not cure (Gatchel et al., 2007). A biopsychosocial perspective takes into account the myriad psychological, social, and contextual factors, in conjunction with biological influences that contribute to the experience, maintenance, and exacerbation of symptoms, as well as response to symptoms and treatments.

In our opinion, a more accurate assessment of the patient may be the key to improve outcome of spine surgery and reduce the rate of Failed Back Surgery Syndrome. An objective evaluation of the psychological profile of the patient can help the selection of the patient for the correct treatment and, at the same time, improve patient’s coping strategies and reduce his/her suffering, which only partially depends on the degenerative modification of the lumbar spine.

### Limitations

The main limitation of the study is the sample size and the relatively short duration of follow up.

Moreover, a larger number of radiological outcome measures could be theoretically included in the analysis. We only considered the Gertzbein-Robbins scale to provide evidence that the surgical procedure has been performed accurately and that persisting disability is not a consequence of screw misplacement.

On the other hand, it should be emphasized that all the patients were treated by the same surgical team in a single center and in a relatively short period of time, thus reducing the confounding factors related to the different context of treatment or surgical technique employed in multicentric studies.

## Conclusion

Pre-operative assessment of psychopathological symptoms in patients with DLSS can predict persistence of disability after LDF for DLSS.

Future studies will evaluate the possibility of modifying post operative outcome through targeted treatment for psychological features emerged during pre-operative assessment.

## Data availability statement

The raw data supporting the conclusions of this article will be made available by the authors, without undue reservation.

## Ethics statement

The studies involving human participants were reviewed and approved by Comitato Etico Regione Autonoma Sardegna. The patients/participants provided their written informed consent to participate in this study.

## Author contributions

EM, DC, and PB: manuscript drafting. GS and GR: conception of the work. GG, FP, EM, and PR: data acquisition. EM and DC: data analysis and interpretation. GS, GR, and VS: critical revision. All authors contributed to the article and approved the submitted version.

## Conflict of interest

EM and GS are consultants for Brainlab AG.

The remaining authors declare that the research was conducted in the absence of any commercial or financial relationships that could be construed as a potential conflict of interest.

## Publisher’s note

All claims expressed in this article are solely those of the authors and do not necessarily represent those of their affiliated organizations, or those of the publisher, the editors and the reviewers. Any product that may be evaluated in this article, or claim that may be made by its manufacturer, is not guaranteed or endorsed by the publisher.
