# Supplementary material for: The role of psychopathological symptoms in lumbar stenosis: A prediction model of disability after lumbar decompression and fusion
Source: Front Psychol. 2023 Mar 22;14:1070205. doi: 10.3389/fpsyg.2023.1070205 (PMC10074599; doi:10.3389/fpsyg.2023.1070205)
Supplement: Supplementary file 1 [file Table_1.DOCX]

**Supplementary materials**

**List of variables analyzed**

Age

Gender

Weight

Height

Body Mass Index

Obesity

Marital status

Job

Hypertension

Diabetes mellitus

Cardiologic comorbidity

Autoimmune comorbidity

Neoplastic comorbidity

Smoke

Previous hip/knee arthroplasty

Previous spine surgery

Level to be treated

Number of level to be treated

Somatization (SCL-90)

Obsessivity (SCL-90)

Interpersonal sensitivity (SCL-90)

Depression (SCL-90)

Anxiety (SCL-90)

Hostility (SCL-90)

Phobic anxiety (SCL-90)

Paranoid ideation (SCL-90)

Psychoticism (SCL-90)

GSI (SCL-90)

PST (SCL-90)

PSDI (SCL-90)

CSI (SCL-90)

Physical functioning (SF-36)

Physical role (SF-36)

Emotional Role (SF-36)

Energy fatigue (SF-36)

Emotional well-being (SF-36)

Social functioning (SF-36)

Pain (SF-36)

General Health (SF-36)

Physical health (SF-36)

Mental Health (SF-36)

Pre-operative Oswestry Disability Index

Visual Analogic Scale Leg Pain

Visual Analogic Scale Back Pain

Days of hospitalization

Pre-operative analgesia

Gertzbein-Robbins scale

Rod lenght

Interbody fusion

Size of interbody fusion

Autologous bone graft

Intra-operative CT

Intra-operative complication

Post-operative complication

**Table 1 - Table containing variable, response and posterior predictive check (PPC) for each patient**

| **Patient** | **Obsessivity** | **Response** | **PPC** |
| --- | --- | --- | --- |
| 1 | 1,00 | 0 | 0,36 |
| 2 | 0,20 | 1 | 0,66 |
| 3 | 1,10 | 1 | 0,33 |
| 4 | 0,30 | 1 | 0,63 |
| 5 | 0,90 | 1 | 0,40 |
| 6 | 2,40 | 0 | 0,06 |
| 7 | 1,50 | 0 | 0,21 |
| 8 | 0,00 | 1 | 0,73 |
| 9 | 2,00 | 0 | 0,11 |
| 10 | 0,00 | 0 | 0,73 |
| 11 | 1,90 | 0 | 0,13 |
| 12 | 1,90 | 0 | 0,13 |
| 13 | 1,90 | 0 | 0,13 |
| 14 | 0,20 | 1 | 0,66 |
| 15 | 0,11 | 1 | 0,69 |
| 16 | 1,30 | 1 | 0,26 |
| 17 | 1,20 | 0 | 0,30 |
| 18 | 1,90 | 0 | 0,13 |
| 19 | 0,90 | 1 | 0,40 |
| 20 | 0,70 | 0 | 0,48 |
| 21 | 1,10 | 0 | 0,33 |
| 22 | 0,70 | 0 | 0,48 |
| 23 | 2,70 | 0 | 0,04 |
| 24 | 0,20 | 1 | 0,66 |
| 25 | 0,10 | 0 | 0,70 |
| 26 | 0,40 | 1 | 0,59 |
| 27 | 0,30 | 1 | 0,63 |
| 28 | 2,30 | 0 | 0,07 |
| 29 | 2,90 | 0 | 0,03 |
| 30 | 0,80 | 1 | 0,44 |
| 31 | 0,00 | 1 | 0,73 |
| 32 | 0,90 | 0 | 0,40 |
| 33 | 2,20 | 1 | 0,08 |
| 34 | 1,10 | 0 | 0,33 |
| 35 | 0,40 | 1 | 0,59 |
| 36 | 1,80 | 0 | 0,14 |
| 37 | 2,00 | 0 | 0,11 |
| 38 | 0,20 | 1 | 0,66 |
| 39 | 1,83 | 0 | 0,14 |
| 40 | 1,30 | 0 | 0,26 |
| 41 | 0,00 | 0 | 0,73 |
| 42 | 0,40 | 0 | 0,59 |
| 43 | 0,20 | 1 | 0,66 |
| 44 | 0,00 | 1 | 0,73 |
| 45 | 2,90 | 0 | 0,03 |
| 46 | 0,00 | 1 | 0,73 |
| 47 | 0,40 | 0 | 0,59 |
| 48 | 0,80 | 1 | 0,44 |
| 49 | 0,10 | 1 | 0,70 |
| 50 | 0,30 | 0 | 0,63 |
| 51 | 0,10 | 0 | 0,70 |
| 52 | 1,50 | 1 | 0,21 |
| 53 | 0,40 | 0 | 0,59 |
| 54 | 2,20 | 0 | 0,08 |
| 55 | 0,50 | 1 | 0,55 |
| 56 | 0,50 | 1 | 0,55 |
| 57 | 0,50 | 1 | 0,55 |
| 58 | 0,40 | 0 | 0,59 |
| 59 | 0,70 | 1 | 0,48 |
| 60 | 0,50 | 1 | 0,55 |
| 61 | 1,90 | 1 | 0,13 |
| 62 | 0,80 | 1 | 0,44 |
| 63 | 0,30 | 1 | 0,63 |
| 64 | 0,00 | 1 | 0,73 |
| 65 | 0,20 | 1 | 0,66 |
| 66 | 1,83 | 0 | 0,14 |
| 67 | 1,20 | 1 | 0,30 |
| 68 | 0,00 | 1 | 0,73 |
| 69 | 0,00 | 1 | 0,73 |
| 70 | 0,40 | 0 | 0,59 |
| 71 | 0,40 | 1 | 0,59 |
| 72 | 0,20 | 1 | 0,66 |
| 73 | 0,10 | 0 | 0,70 |
| 74 | 0,00 | 1 | 0,73 |
| 75 | 0,00 | 1 | 0,73 |
| 76 | 0,30 | 1 | 0,63 |
| 77 | 0,50 | 1 | 0,55 |
| 78 | 0,30 | 1 | 0,63 |
| 79 | 1,50 | 0 | 0,21 |
| 80 | 0,10 | 1 | 0,70 |
| 81 | 0,60 | 0 | 0,51 |
| 82 | 0,00 | 1 | 0,73 |
| 83 | 1,20 | 0 | 0,30 |
| 84 | 0,70 | 0 | 0,48 |
| 85 | 0,20 | 0 | 0,66 |
| 86 | 1,70 | 0 | 0,16 |
| 87 | 0,90 | 0 | 0,40 |
| 88 | 1,60 | 0 | 0,18 |
| 89 | 0,20 | 1 | 0,66 |
| 90 | 1,70 | 0 | 0,16 |
| 91 | 0,90 | 0 | 0,40 |
| 92 | 0,20 | 0 | 0,66 |
| 93 | 0,00 | 1 | 0,73 |
| 94 | 1,60 | 0 | 0,18 |
| 95 | 0,60 | 0 | 0,51 |
| 96 | 0,90 | 0 | 0,40 |
| 97 | 0,10 | 1 | 0,70 |
| 98 | 1,10 | 1 | 0,33 |
| 99 | 1,00 | 0 | 0,36 |
| 100 | 1,30 | 0 | 0,26 |
| 101 | 0,20 | 0 | 0,66 |
| 102 | 0,40 | 0 | 0,59 |
| 103 | 0,50 | 0 | 0,55 |
| 104 | 2,10 | 0 | 0,10 |
| 105 | 1,50 | 0 | 0,21 |
| 106 | 0,90 | 1 | 0,40 |
| 107 | 1,20 | 1 | 0,30 |
| 108 | 1,00 | 0 | 0,36 |
| 109 | 0,00 | 0 | 0,73 |
| 110 | 0,20 | 0 | 0,66 |
| 111 | 1,20 | 0 | 0,30 |
| 112 | 0,60 | 1 | 0,51 |
| 113 | 1,20 | 1 | 0,30 |
| 114 | 0,70 | 1 | 0,48 |
| 115 | 0,90 | 0 | 0,40 |
| 116 | 1,90 | 0 | 0,13 |
| 117 | 0,70 | 0 | 0,48 |
| 118 | 0,90 | 0 | 0,40 |
| 119 | 0,80 | 1 | 0,44 |
| 120 | 1,50 | 0 | 0,21 |
| 121 | 0,30 | 0 | 0,63 |
| 122 | 1,20 | 0 | 0,30 |
| 123 | 0,90 | 0 | 0,40 |
| 124 | 1,00 | 0 | 0,36 |
| 125 | 0,50 | 1 | 0,55 |
